# Spontaneous spinal epidural hemorrhage due to dengue fever causing hemiparesis in a non-endemic zone: A case report

**DOI:** 10.5339/qmj.2025.119

**Published:** 2025-12-14

**Authors:** Sabyasachi Ghosh, Fatma Jassim Al Kuwari, Shrijeeta Ghosh

**Affiliations:** 1 Hamad Medical Corporation, Doha, Qatar *Email: sghosh@hamad.qa

**Keywords:** Dengue, spontaneous spinal epidural hemorrhage, hemiparesis, Qatar

## Abstract

**Background:**

Dengue is the most common arthropod-borne viral illness in humans and is prevalent in tropical and subtropical regions worldwide. Nearly half of the world’s population living in these endemic areas is at risk of infection.

**Case presentation:** A 42-year-old Bangladeshi male, previously healthy and working as a laborer, was admitted to Hazm Mebaireek General Hospital—a Qatar government hospital serving mainly laborer populations—with a 3-day history of abdominal pain, fever, and intermittent vomiting. He had no recent history of travel to Bangladesh before this incident. Viral studies were positive for dengue virus immunoglobulin G and immunoglobulin M. Magnetic resonance imaging revealed a posterior extradural collection extending from cervical (C) 3 to dorsal (T) 7, resulting in compression of the posterior aspect of the thecal sac and causing it to appear off-center within the spinal canal. The findings suggested that the collection was most likely infective in origin. He underwent a right-sided C7 hemilaminectomy and evacuation of an epidural hematoma at Hamad General Hospital, Qatar. Postoperatively, he improved significantly. He was subsequently transferred to the Qatar Rehabilitation Institute for an active rehabilitation program and was discharged upon completion of rehabilitation.

**Discussion:** Dengue is prevalent in subtropical regions and South America, accounting for nearly 75% of global cases. Qatar is a non-endemic zone for dengue fever. Spinal cord epidural hematoma due to dengue fever is extremely rare, with only five cases reported to date according to our literature review. Our case will be the 22nd reported instance—and the sixth among similar series—of spontaneous spinal hemorrhage due to dengue in the literature, and the first reported from a non-endemic zone. The neurological manifestation in our case was hemiplegia. Among the five previously reported cases, two presented with quadriplegia and three with paraplegia.

**Conclusion:**

Gulf countries are at particularly high risk among non-endemic regions due to the large number of expatriates returning from endemic areas. This case indicates that atypical presentations of dengue can also occur in non-endemic regions. We believe that early screening for tropical fevers in suspected cases can facilitate prompt diagnosis and management of dengue fever. To our knowledge, this is the first reported case of spinal hemorrhage due to dengue fever from a non-endemic zone. Moreover, our patient presented with hemiparesis, whereas the four previously reported cases manifested as either quadriplegia or paraplegia.

## 1. INTRODUCTION

Dengue is the most common arthropod-borne viral (arboviral) illness in humans. It is caused by the Dengue virus (DENV), a single-stranded ribonucleic acid (RNA) virus, and is transmitted by the mosquito Aedes aegypti. Dengue is prevalent in tropical and subtropical regions worldwide, placing approximately half of the world’s population at risk.^1,2^ In 2023, over 80 countries of the WHO reported a historic high of more than 6.5 million cases and over 7,300 deaths due to dengue.^3^ There are four serotypes of dengue fever. Infection with one serotype confers lifelong immunity to that specific serotype, but individuals remain susceptible to the other three.^4^

Qatar is a small country with a population of approximately 3.12 million and is considered a non-endemic region for dengue. Expatriates constitute 88% of the population, most of whom come from dengue-endemic countries. Gulf countries, including Qatar, are at relatively higher risk among non-endemic regions due to the continuous influx of expatriates from endemic areas throughout the year.^5^ Qatar has a well-developed medical infrastructure supported by both public and private organizations. Hazm Mebaireek General Hospital, where the patient was initially admitted, and Qatar Rehabilitation Institute, where the patient received rehabilitation care, are highly advanced public medical facilities under Hamad Medical Corporation (HMC), the principal public healthcare provider in the country.^6^

This is a case report. Informed consent for publication was obtained from the patient, and the report was approved by the HMC Medical Research Center (MRC-04-24-821).

## 2. CASE PRESENTATION

A 42-year-old Bangladeshi male worker, previously healthy, drove himself to the Hazm Mebaireek General Hospital Emergency Department in late 2023. He reported a 3-day history of abdominal pain, fever, and intermittent vomiting, with no recent travel history. On admission, his abdomen was soft and lax, with non-specific lower abdomen tenderness. There was no testicular pain or skin rash, and rectal examination revealed no black stool. Examination of the lower limbs showed no edema, with intact perfusion and pulses. He was conscious, alert, and oriented, with no meningeal signs. The patient also complained of upper back and neck pain. He was ataxic, prone to falls, and unable to walk independently. Differential diagnoses included abdominal sepsis, intestinal obstruction, hemophagocytic lymphohistiocytosis (HLH), thrombotic thrombocytopenic purpura (TTP), hepatitis, and tropical infection. Complete blood tests were ordered, and the results are presented in Table 1.

**Figure 1:**
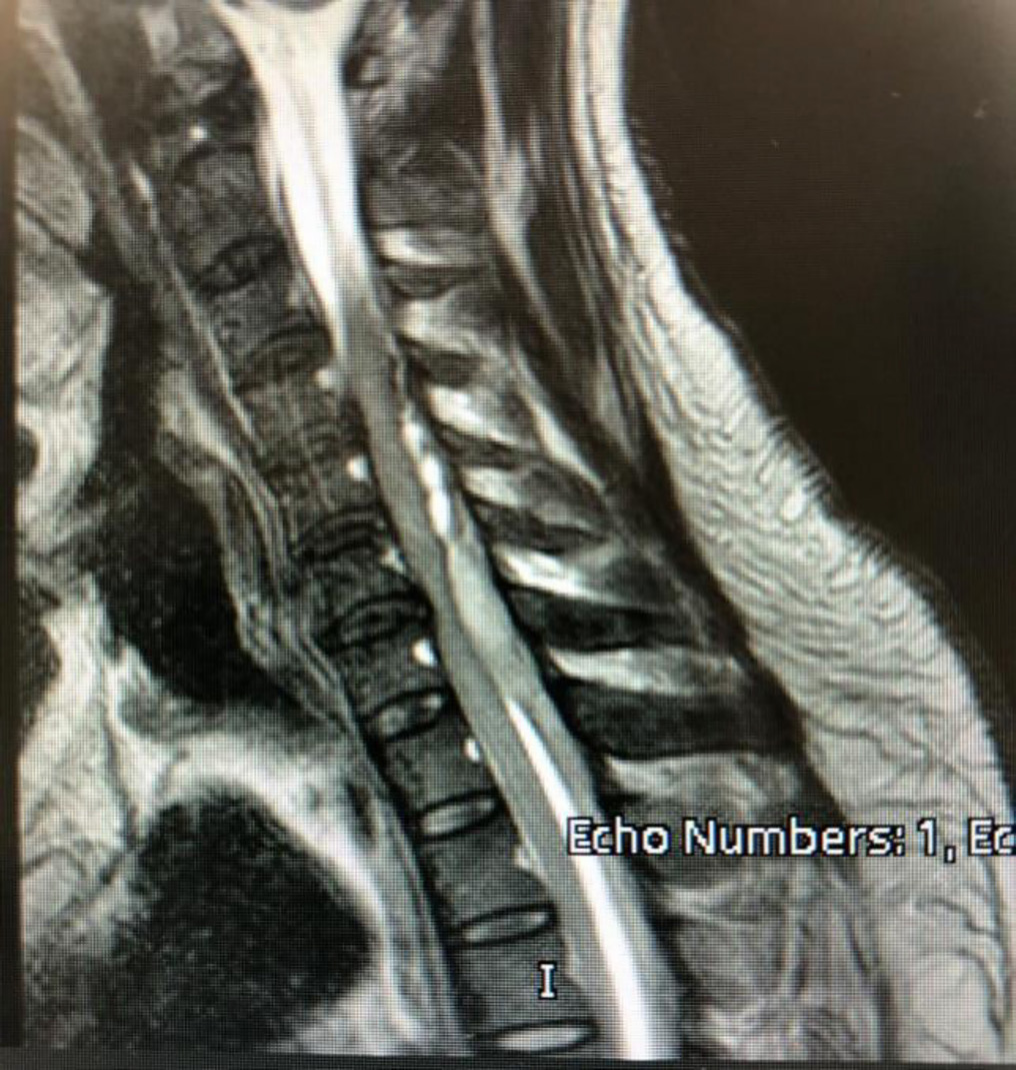
MRI showing a posterior extradural collection.

An abdominal ultrasound was performed and showed moderate fatty liver changes and acalculous cholecystitis. A computed tomography (CT) of the abdomen also revealed fatty liver changes associated with mild to moderate ascites, without evidence of bowel obstruction, ischemia, or perforation. Magnetic resonance imaging (MRI) performed two days after admission showed mild peripheral enhancement in the lower cervical and upper thoracic regions, with a posterior extradural collection extending from cervical(C) 3 down to dorsal(D) 6, as shown in Figure 1. The extradural collection reached its maximum anteroposterior dimension of 8 mm at the C6/C7 level. The resultant compression of the posterior aspect of the thecal sac appeared eccentric and off-center within the spinal canal. No significant signal changes within the spinal cord were noted. The findings suggested that the collection was most likely infective in origin.A viral panel performed for two days post-admission was positive for dengue virus immunoglobulin G (IgG) and immunoglobulin M (IgM). Tests for Brucella antibodies (IgG and IgM), hepatitis A antibody (IgM), hepatitis B surface antigen, hepatitis C antibodies, hepatitis E virus IgG and IgM, human immunodeficiency virus (HIV) antigen/antibody combo, and COVID-19 polymerase chain reaction (PCR) were all negative. The patient was evaluated by hematologists, and platelet transfusions were initiated—two units twice daily—to correct thrombocytopenia, with a target platelet count above 50 per milliliter of blood. On day 3 post-admission, he was transferred from Hazm Mebaireek General Hospital to Hamad General Hospital for neurosurgical intervention. Hamad General Hospital is a Joint Commission International (JCI)-accredited multispecialty hospital under HMC.

On admission to the neurosurgery unit at Hamad General Hospital, his Glasgow Coma Scale score was 15. Motor strength on the left side was normal (with the Medical Research Council [MRC] scale 5/5), while he exhibited right-sided hemiparesis. Muscle power on the right upper limb was 3/5 at the shoulder, 2/5 at the elbow, and 0/5 at the hand, and the right lower limb had 0/5 power. Sensation was intact in all four limbs. The Hoffman sign was positive on the right, and the right plantar response was upward, consistent with an upper motor neuron lesion.

He was unable to walk due to right-sided weakness and required a Foley catheter for urinary retention. On day 2 post-admission to Hamad General Hospital, he underwent a right C7 hemilaminectomy with evacuation of an epidural hematoma, resulting in significant postoperative improvement. On day 8, he was transferred from Hamad General Hospital to the Qatar Rehabilitation Institute to undergo an active rehabilitation program. On admission to the Qatar Rehabilitation Institute, the patient was vitally stable, afebrile, oriented, and cooperative. Higher mental functions and cranial nerves were intact. He was continent for both bladder and bowel, and his sensory system was intact. Range of motion, muscle tone, and coordination were within normal limits. His muscle strength was as follows: upper limbs 5/5, left lower limb 5/5, and right lower limb 3+/5. Sitting and static standing balance were good, while his dynamic standing balance was fair. Bed mobility was independent, sit-to-stand was modified independent, and bed-to-chair transfers were supervised. He was able to walk indoors on level surfaces for 150 m with a minimal ataxic gait pattern, requiring one-person contact guard assistance, and needed minimal assistance for ascending and descending stairs.

He was completely independent in eating, grooming, and upper-body dressing. He was modified independent in toileting, lower-body dressing, and toilet/shower transfers. He required setup/supervision for bathing. His functional independent measure (FIM) score was 110/126, and his mini-mental state examination (MMSE) score was 30/30. The patient was discharged from the Qatar Rehabilitation Institute after 12 days of active rehabilitation and 20 days after his admission to Hamad General Hospital. On discharge from the Qatar Rehabilitation Institute, the patient was vitally stable, afebrile, oriented, and cooperative. His cranial nerves and higher mental functions were intact. His mood and sleep were normal, and his appetite was good. He was continent of both bladder and bowel. His sensory system was intact, and his range of motion, muscle tone, and coordination were within normal limits. Muscle strength was 5/5 in both upper limbs and the left lower limb, and 4+/5 in the right lower limb. His sitting and standing balance were good. Bed mobility was independent, sit-to-stand was modified independent, and transfers from bed to chair were independent. He was able to walk indoors and outdoors with a slow gait, primarily due to caution. He was able to ascend and descend stairs with modified independence using bilateral handrails. He was completely independent in eating, grooming, upper-body dressing, toileting, and shower transfers. He was modified independent in bathing and lower-body dressing. His outcome measures were as follows: FIM score of 122/126, MMSE score of 30/30, and a disability scale score of 2.

## 3. DISCUSSION

The two most severe sequelae of dengue infection are dengue hemorrhagic fever and dengue shock syndrome.^7^ Neurological manifestations were initially reported as atypical features of dengue infection in 1976.^8^ Neurological involvement may occur in 0.5%–20% of dengue fever cases.^9^ Overall, less than 1% of systemic complications in dengue are due to neurological manifestations.^10^ Numerous neurological manifestations, including headaches, seizures, behavioral disturbances, and altered sensorium, may occur as part of encephalitis or aseptic meningitis. Mononeuropathies, polyneuropathies, Guillain–Barré syndrome, myelitis, thrombosis, and intracranial hemorrhage were also reported in patients with dengue fever.^10,11^ Additionally, quadriparesis due to myositis, Guillain–Barré syndrome, and hypokalemia^12,13^ has been reported.

Dengue is prevalent in subtropical regions and South America, accounting for approximately 75% of global cases. Qatar, however, is a non-endemic zone for dengue fever.^4^ Spontaneous spinal cord hemorrhage is extremely rare in dengue, with only five cases reported to date, according to our literature review.^14–18^ Our case represents the sixth reported instance of spontaneous spinal hemorrhage due to dengue in the literature and the first case documented from a non-endemic region. Brief descriptions of the previously reported five cases are presented chronologically in Table 2.

To investigate the cause of dengue fever in a non-endemic country, we obtained a detailed history of the patient’s recent travel. The patient reported being sent to an island in Qatar for construction work. Seven days before the patient developed symptoms, numerous expatriate workers were present at the work site, which contained a tank with stagnant water conducive to mosquito breeding. Many of these workers were from dengue-endemic areas and could have entered Qatar with subclinical dengue infections. The local mosquitoes likely acted as vectors, transmitting the virus to coworkers, including our patient.To explain this rare occurrence in our patient, we postulate that the patient may have previously contracted dengue fever while living in Bangladesh, an endemic region. After several years in Qatar, exposure to a different dengue serotype could have triggered this hemorrhagic manifestation through antibody-dependent enhancement of the immune response.^4^ In our case, the neurological manifestation was hemiplegia. Among the five previously reported cases, two presented with quadriplegia and three with paraplegia. This suggests that while the underlying etiology—spinal hemorrhage due to dengue—remains the same, the neurological presentation can vary.

A limitation of this case report is that the patient was from a dengue-endemic region. Consequently, such an occurrence is less likely to occur in non-endemic countries with a lower number of migrant workers from dengue-endemic zones.

## 4. CONCLUSION

This case highlights that atypical presentations of dengue can occur in non-endemic regions through carriers from endemic areas. To our knowledge, this is the first reported case of spinal hemorrhage due to dengue fever from a non-endemic zone. Notably, our patient presented with hemiparesis, whereas previously reported cases involved quadriparesis or paraparesis. He underwent a right-sided C7 cervical hemilaminectomy with evacuation of epidural hematoma and postoperatively showed dramatic improvement.

In conclusion, clinicians should consider spontaneous spinal cord hemorrhage as a potential cause of neurological deficits—such as paraparesis, quadriparesis, or hemiparesis—in patients with dengue. Early recognition and timely management are essential for achieving favorable outcomes.

## COMPETING INTERESTS

The authors have no conflicts of interest to declare.

## Figures and Tables

**Table 1.** Key laboratory findings at the time of patient admission.

**Test**	**Normal range**	**Result**	**Comment**
C-reactive protein	<3 mg/L (milligram per liter)	33.6 mg/L	High
Bilirubin total	<0–21 umol/L (micromole per liter)	44 umol/L	High
Bilirubin direct	<5.1 umol/L	25 umol/L	High
Alanine transaminase	0–1 U/L (units per liter)	975 U/L	High
Aspartate aminotransferase	8–33 U/L (units per liter)	2,517	High
Lactate dehydrogenase	0–40 U/L (units per liter)	1,800 U/L	High
Platelet	150–410×10^3^/uL (per microliter)	49×10^3^/uL	Low
International normalized ratio (INR)	<1.1	1.8	Mildly high

**Table 2.** Previously reported cases of spontaneous spinal hemorrhage due to dengue.

**Number/year of publication**	**Country**	**Gender/age**	**Main neurological manifestation**	**Lesion**	**Outcome**
1. Year 2010^14^	India	Male/40 years	Quadriparesis	C3–C4 extradural acute hemorrhage	Complete recovery after a month of discharge
2. Year 2017^15^	Malaysia	Female/37 years	Paraparesis	Spinal subarachnoid hemorrhage at T4–T9 level	Paraparesis did not improve
3. Year 2019^16^	India	Female/66 years	Quadriparesis	Cervical and dorsal acute subarachnoid hemorrhage	Deceased on 13th day of illness
4. Year 2022^17^	India	Female/48 years	Paraplegia	Spontaneous intradural hematoma D7–D8	Marginal improvement of paraplegia at 1-year follow-up
5. Year 2022^18^	India	Female/54 years	Paraparesis	Spinal subarachnoid	Complete recovery after
				hemorrhage at T12–L1 level	2 months
